# Human amniotic membrane plug to promote failed macular hole closure

**DOI:** 10.1038/s41598-020-75292-2

**Published:** 2020-10-26

**Authors:** Tomaso Caporossi, Bianca Pacini, Daniela Bacherini, Francesco Barca, Francesco Faraldi, Stanislao Rizzo

**Affiliations:** 1 Department of NEUROFARBA, Ophthalmology, University of Florence, Largo Brambilla 3, Careggi, 50134 Florence, Italy; 2 Ospedale Oftalmico Di Torino, Turin, Italy; 3 Ophthalmology Unit, Fondazione Policlinico Universitario A. Gemelli IRCCS, 00196 Rome, Italy; 4 Department of Ophthalmology, Catholic University of “Sacro Cuore”, 00168 Rome, Italy

**Keywords:** Diseases, Medical research

## Abstract

The failed macular hole is a full-thickness defect involving the fovea that fails to close despite 1 or more surgeries. While many surgical options have been proposed to manage it, none of these guarantee complete anatomical success and satisfactory visual recovery. We report postoperative outcomes on 36 patients affected by failed macular hole, treated with a human amniotic membrane plug transplant. Follow-ups were performed with a standard ophthalmological examination and with advanced multimodal diagnostic imaging. Anatomical closure was achieved at 3 months in all patients. Mean best-corrected visual acuity improved statistically significantly at 6 months (*p* < 0.05). Through microperimetric tests, we assessed a partial recovery of the macular sensitivity on the edges of the plug. Analyzing SD-OCT images, we reported a tissutal ingrowth above the plug, and its segmentation into layers, mimicking normal retinal architecture. OCT-Angiography images non invasively analysed the retinal parafoveal capillary microvasculature; the elaboration of Adaptive Optics images showed the presence of photoreceptors at the edges of the plug. This work demonstrates not only the complete anatomical success of our technique, but also remarkable functional results, and opens the door to a greater understanding of modifications induced by the presence of a human amniotic membrane plug.

## Introduction

Pars plana vitrectomy (PPV) with internal limiting membrane (ILM) peeling after staining with dyes, gas tamponade, and facedown positioning of the patient is the standard technique for treating a macular hole (MH), with reports of primary closure rates exceeding 90%^1^.

Inverted ILM flap is a very successful technique in the case of large MHs or myopic MHs, as first described in 2010 by Michalewska et al.; it improved their closure rate to 98%^2^.

Despite the high anatomical success rate, previous studies have shown an incidence of around 3.3% of MH reopening when small-gauge vitrectomy is used. Risk factors for MH reopening include high myopia, residual epiretinal traction, insufficient gas tamponade, and insufficient face-down positioning^3–6^. Recurrent or persistent MH treatment is still a challenge for a vitreoretinal surgeon, as traditional techniques cannot be used for eyes subjected to a previous PPV with complete ILM peeling.

Several new techniques have been described to increase the anatomical and functional outcomes in these cases. The modern approach to closing a failed macular hole (FMH) is to insert alternative tissues into the macular hole, to promote their anatomical closure and the improvement of visual acuity. While many surgical options have been proposed, none of these guarantee complete anatomical success and satisfactory visual recovery; moreover, these alternative techniques are often burdened by limits related to the technical complexity of execution, the need for 2 surgical times, or the need for tissues that are not always readily available.

The use of the human amniotic membrane (hAM) patch to close 8 recurrent macular holes was already proposed by Rizzo et al. in 2018^7^; the anatomical success was 100% with a mid-term follow-up (6 months after surgery) and 20% sulfur hexafluoride (SF_6_) used as the endotamponade in all cases. The use of the hAM plug has also been proven to be effective in repairing retinal breaks^7^, large macular tears associated with retinal detachment^8^, and high-myopic macular holes associated with retinal detachment^9^.

This work aims to report the anatomical and functional outcomes in a large series of patients affected by FMH, treated using a hAM plug; we also performed a wide series of instrumental analyses, including SD-OCT, OCT-Angiography (OCT-A), Microperimetry, and Adaptive Optics (AO), to evaluate the anatomical and functional results of this technique and to better understand the modifications induced by the hAM plug.

## Results

Thirty-six eyes of 36 patients with FMH who had already undergone PPV with complete ILM peeling were included in this study. Fifteen were male and 19 were female; the mean age was 66.3 ± 12.3 years (range 37–84 years). Seventeen patients were high-myopic (with axial length > 26.5 mm) with a basal best-corrected visual acuity (BCVA) of 0.96 ± 0.25 logMAR (range 1.3–0.5 logMAR), with MH maximum diameter of 860 ± 380 μ (range 402–1640 μ) and MH minimum diameter of 672 ± 281 μ (range 308–1520 μ). The non-high-myopic group contained 19 patients with a basal BCVA of 1.34 ± 0.45 logMAR (range 0.7–2 logMAR), and with MH maximum and minimum diameter of 1140 ± 373 μ (range 538–1810 μ) and 675 ± 167 μ (range 467–1000 μ), respectively. Five patients—3 in the high-myopic group (17.6%) and 2 in the non-high-myopic group (10.5%)—received 2 or more surgeries before recruitment. Table 1 summarizes patient demographics and baseline clinical data.

**Table 1:** Demographics and baseline clinical data.

Variables	Total group (n = 36)	High myopia (n = 17)	Non-high myopia (n = 19)
Male–Female, No	15–19	5–12	12–7
Age, mean (SD), years	66.3 ± 12.3	64.7 ± 10.8	67.7 ± 13.1
BCVA, mean (SD), logMAR	1.15 ± 0.14	0.96 ± 0.25	1.34 ± 0.45
MH maximum diameter, mean (SD), µm	1007 ± 397	860 ± 380	1140 ± 373
MH minimum diameter, mean (SD), µm	674 ± 224	672 ± 281	675 ± 167
≥ 2 surgeries, No. (%)	5 (13.8)	3 (17.6)	2 (10.5)
Phakic, No. (%)	8 (22.2)	5 (29.4)	3 (15.7)

Patients of the study underwent 23-gauge PPV and hAM plug transplantation at the Eye Clinic of Careggi University Hospital (Florence, Italy). Eight patients were phakic, while 5 (29.4%) and 3 (15.7%) were in the high-myopic and non-high-myopic groups, respectively, and underwent phacoemulsification and IOL implantation combined with PPV. Patients were examined at baseline and at 2 weeks and 1, 3, and 6 months after surgery with standard ophthalmological examinations and SD-OCT; at 6 months, they also underwent OCT-A, Microperimetry, and Adaptive Optics.

Two patients left after the 1-month follow-up.

### Best-corrected visual acuity

Mean 6-month BCVA improved from 1.15 ± 0.14 logMAR to 0.65 ± 0.26 logMAR (range 1.3–0.2 logMAR), which was statistically significant (*p* = 0.00000017).

In the high-myopic group, mean postoperative BCVA improved from 0.96 ± 0.25 logMAR to 0.61 ± 0.13 logMAR (ranging from 0.4 to 0.8 logMAR) and the difference was statistically significant (*p* = 0.0005). All patients but 3 gained 1 or more Snellen lines. One patient lost 2 Snellen lines and 2 patients remained stable.

In the non-high-myopic group, mean postoperative BCVA improved from 1.34 ± 0.45 logMAR to 0.58 ± 0.24 logMAR (range 1–0.3 logMAR) and the difference was statistically significant (*p* = 0.000001). All patients gained 1 or more Snellen lines.

### Optical coherence tomography

One hundred percent FMH was found closed at the OCT scans. At the 2-week follow-up, complete macular hole closure was found in 33 of the 36 patients (91.6%). In 2 patients (5.5%) with non-myopic FMH, tamponed with air, OCT scans showed a not-complete closure of the MH at 2 weeks, with the margins of the hole nestling over the well-placed hAM plug. These 2 patients did not receive further surgery or endotamponade injection. The 3-month OCT showed complete closure of the FHM in both cases. One patient affected by a failed high-myopic macular hole had a hAM plug dislocation and needed a second surgery with a new hAM plug transplantation. Once the plug was replaced, the hole was found to be closed at the first follow-up.

### Optical coherence tomography angiography

OCT-A (AngioVue Optovue, Fremont, CA) was used to analyze the vascular density (VD) of the superficial capillary plexus (SVD), the deep capillary plexus (DVD), and the foveal avascular zone (FAZ), which were calculated using the built-in software.

**Figure 1:**
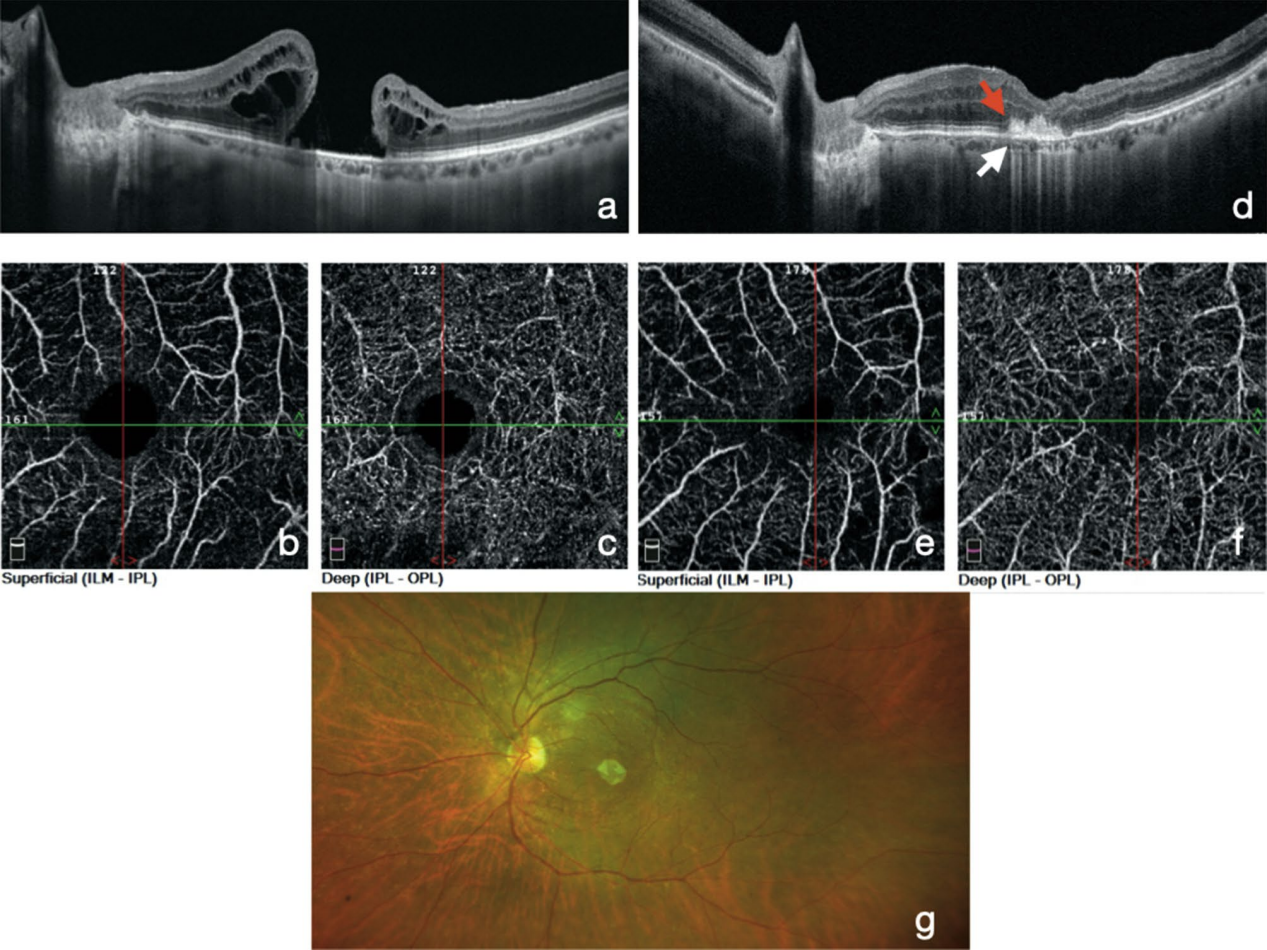
SD-OCT and OCT-Angiography results in failed macular hole treated with hAM. Panels **a**–**c** represent preoperative structural OCT (**a**) and OCT-A (**b**,**c**) analysis of a failed macular hole. Panels **d**,**f** represent 6-month postoperative analysis of the same patient. (**d**) structural OCT shows the hAM plug well-positioned under the retina (white arrow) and the macular hole appears completely closed. On the nasal side, a partial reconstruction of the outer layers of the retina appears over the hAM plug (red arrow). (**e**,**f**) OCT-A shows a reduction of the dimensions of the FAZ area compared to preoperative analysis, as well as a normal qualitative texture of the superficial and deep capillary plexus. A wide-field color fundus photograph (**g**) shows a well-positioned round-shape hAM patch in the macular area.

The scanning area was captured in 3 × 3 mm sections centered on the fovea. The images were acquired at the 6-month follow-ups (Fig. 1).

In the non-high-myopic group, SVD was 37.7 ± 9.33% (range 15–52%) and 44.63 ± 5.7% (range 32–53.2%) for treated and fellow eyes, respectively, and the difference was statistically significant (*p* = 0.01). DVD was 47 ± 3.9% (range 41.7–52%) and 50.12 ± 7.68% (range 25.6–58.8%) for treated and fellow eyes, and the difference was not statistically significant (*p* = 0.12).

In the high-myopic group, SVD and DVD were 29.5 ± 9% (range 15–42%) and 36.8 ± 7.1% (range 26–50.4%) for treated eyes, and 40.8 ± 6.8% (range 27–49.1%) and 48 ± 12% (range 20.5–59.7%) for fellow eyes. The difference was statistically significant for both SVD and DVD (*p* = 0.002, *p* = 0.01).

The FAZ area was 0.39 ± 0.32 mm^2^ and 0.54 ± 0.62 mm^2^ for treated and fellow eyes, and the difference was not statistically significant (*p* = 0.13).

In the non-high-myopic group, FAZ was 0.36 ± 0.22 mm^2^ and 0.49 ± 0.6 mm^2^ for treated and fellow eyes, and the difference was not statistically significant (*p* = 0.22).

In the high-myopic group, FAZ was 0.43 ± 0.42 mm^2^ and 0.6 ± 0.6 mm^2^ for treated and fellow eyes, respectively, and the difference was not statistically significant (*p* = 0.21).

We found no statistically significant difference between the data of patients tamponed with gas and those of patients tamponed with air. The results of the statistical analysis are summarized in Tables 2 and 3.

**Table 2:** Comparisons between OCT-A parameters of treated eyes and fellow eyes.

Parameters	Total group		High myopic		Non-high myopic	
	Treated eye	Fellow eye	Treated eye	Fellow eye	Treated eye	Fellow eye
SVD (%)	34.26*	43.14	29.58*	40.84	37.78*	44.63
DVD (%)	42.2*	49.3	36.8*	48.03	47	50.12
FAZ (mm^2^)	0.39	0.54	0.43	0.60	0.36	0.49

*SVD* superficial vascular density, *DVD* deep vascular density, *FAZ* foveal avascular zone.

*p < 0.05.

**Table 3:** Correlations coefficient between final visual acuity and OCT-A parameters.

Parameters	Total group	High-myopic	Non high myopic
SVD	− 0.25	− 0.35*	− 0.22
DVD	− 0.25	− 0.26	− 0.25
FAZ	− 0.03	− 0.23	0.01
Maximum diameter	− 0.28	− 0.37*	− 0.29
Minimum diameter	− 0.08	− 0.45*	0.25

*SVD* superficial vascular density, *DVD* deep vascular density, *FAZ* foveal avascular zone.

*High correlation.

### Microperimetry

Analysing the macular sensitivity maps obtained using Microperimetry, we noticed a partial recovery of the macular sensitivity at the edges of the hAM plug; in all patients examined, the fixation shifted to the periphery of the macula, on the border of the hAM plug, where the Microperimetry showed an increased sensibility.

### Adaptive optics

**Figure 2:**
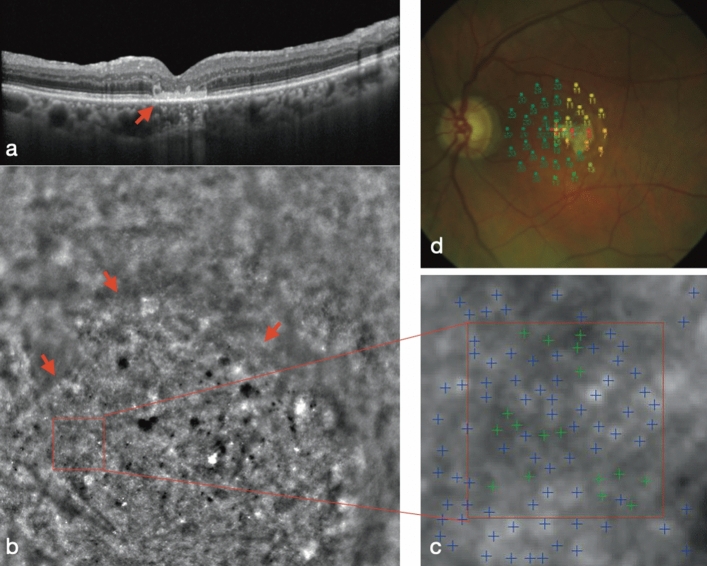
Adaptive optics and microperimetry results. (**a**) A 6-month postoperative structural OCT shows macular hole closure and the subretinal hAM plug (red arrow). (**b**) Images from the Rtx1 adaptive optics (AO) fundus camera: the red square represents the area investigated using the Rtx1 AO fundus camera. The red arrows indicate the edge of the patch. The magnified image of the area indicates some bright dots corresponding to photoreceptors. (**c**) The automatically-processed zone (red square in fig. **b**) of the AO image shows the photoreceptors (blue cross) detected by the Rtx1 software. (**d**) Retinal sensitivity map obtained using microperimetry: a central small area of absolute scotoma (red circles) corresponds to a lack of sensitivity, while a surrounding wider area of the relative scotoma (orange and yellow circles) indicates a preservation of retinal sensitivity over the hAM plug.

Adaptive Optics images were acquired at 2° from the fovea along the 4 meridians (nasal, temporal, superior, and inferior) both in treated eyes and in fellow eyes. Flood-illumination camera Rtx1 images showed a defocus effect at the interface of the peripheral retina/hAM plug, due to a variation in the orientation of the external retinal margin over the plug. Outside the margin of the plug, we detected the presence of hyper-reflective dots; similar fine structures could be seen in correspondence to the hAM plug, especially at the border. The integrated software (AO Detect Mosaic) can distinguish the presence of these hyper-reflective dots and interpreted them as photoreceptors. Some artifacts, due to the irregularity of the transplant surface, were observed in the fovea. Some bigger hypo-reflective dots were found in the center and on the edge of the plug; the software did not recognize them as photoreceptors (Fig. 2). The mean cone density in the superior quadrant was 9144 ± 5310.372; in the temporal quadrant it was 6484.5 ± 1672.308, in the nasal quadrant it was 14,387 ± 3623.215, and in the inferior quadrant it was 6449 ± 3811.306 at 2° eccentricity. Inter-cone spacing in the superior quadrant was 12 ± 2.82 μm; in the temporal quadrant it was 12.5 ± 0.7 μm, in the nasal quadrant it was 9 ± 1.41, and in the inferior quadrant it was 13.5 ± 3.53 μm at 2° eccentricity. A comparison of the control eyes revealed that cone density was significantly reduced in all 4 quadrants at 2° and that spacing between the cones was significantly increased at 2° in all 4 quadrants at 2° eccentricity.

### Safety

No adverse events, such as an increase in intraocular pressure (IOP) or endo-ocular inflammation, were reported during the follow-up period. No rejection of the hAM implanted in the subretinal space was reported, nor was an inflammation reaction such as intraretinal macular oedema or epiretinal membrane development, or major complications such as retinal detachment or endophthalmitis.

## Discussion

The treatment of failed macular holes is still a challenge for a vitreoretinal surgeon. Many new techniques for managing FMH have been described; among the most modern approaches, the peeling of the ILM from the periphery of the posterior pole^10^ and neurosensory retinal transplantation^11^ have proved successful.

In our study, 100% of FMH were found to be closed at the OCT scans. Thirty-three FMH out of 36 (91.6%) were found to be closed at 2 weeks; 35 FMH out of 36 (97.22%) were found closed at 3 months; and only 1 patient needed additional hAM transplant surgery, likely related to the learning curve of the technique, which led to incorrect placement of the hAM plug with the epithelial layer instead of the chorion layer facing the RPE. Once the plug was replaced, the hole was found closed at the first follow-up.

Grewal et al. treated 41 eyes of 41 patients with FMH with an autologous retinal transplant and reported a final FMH closure of 36 eyes out of 41 (87.8%)^12^. We achieved 97.22% FMH closure after 1 surgical operation and 100% after 2 operations. In Grewal's series, 28 eyes were highly myopic, and in 25 out of 28 anatomic closure was achieved (89.3%). In our study, in the highly-myopic group, we reached a final closure rate of 100% after 2 hAM transplant operations, and of 94.11% after only 1 operation.

Morizane et al. treated 10 FMH eyes with a 90% closure rate using autologous ILM transplants. In their series, they treated 2 highly-myopic eyes with a closure rate of 100%^10^. Lee et al. have described a proliferation of glial tissues, foveal fibroplasia, and depigmentation in the macular area after ILM autologous transplantation for FMH, which can affect final visual recovery^13^. In our series, OCT scans during the follow-up period showed tissue ingrowth above the hAM plug, and a formation of layers inside it, especially at the level of the external retina (Fig. 1).

In our series, 33 eyes out of 36 (91.6%) experienced improved BCVA, while 3 patients (8.4%) had stable or worsening BCVA. Y. Morizane et al. reported a BCVA increase from 0.99 ± 0.25 to 0.57 ± 0.36 logMAR, with 9 patients out of 10 obtaining an increase of at least 2 Snellen lines^10^.

Grewal et al. reported that 36 eyes treated with autologous retinal transplant had complete anatomic closure of the FMH: 12 patients (33.3%) with stable BCVA, 19 patients (52.3%) with improved BCVA, and 5 patients (13.8%) with worsening BCVA. Mean BCVA improved from 1.11 ± 0.66 logMAR to 1.03 ± 0.51 logMAR^12^. In this study, visual improvement was defined as an increase of at least 0.3 logMAR units, and decline was defined as a decrease of at least 0.3 logMAR units. Following these parameters, in our study, we found an improvement of BCVA in 69.4% of our patients, none of whom suffered BCVA deterioration.

In non-high-myopic patients, OCT-A showed no statistical difference in deep vascular density (DVD) between treated eyes and fellow eyes, while the superficial vascular density (SVD) showed a statistically different lower density compared to fellow non-treated eyes. These data concerning DVD are in contrast to those of previous studies on OCT-A in idiopathic MH treated with ILM peeling showing a reduced VD in deep plexus after surgery^14,15^. Our results on DVD suggest that in non-myopic eyes, the effect of the hAM plug is more evident at the level of the anatomical parts to which it is more attached, influencing mostly the deep capillary plexus with a trophic-like effect, thus reducing the VD differences between the treated eye and the fellow not-treated eye. Interestingly, we found no statistical differences in the FAZ of both groups of treated eyes, compared to that of fellow eyes; this result is not in line with those of previous studies on idiopathic MH operated with ILM peeling^14,16,17^, in which a remarkable reduction in the FAZ is always reported. In light of this, we can hypothesize that the hAM plug does not induce a centripetal movement of the tissues, which can lead to a reduction in the foveal area, as has been instead supposed for ILM peeling^14,17^. Our data revealed that the increase in visual acuity is moderately related to increased vascularization of the parafoveal segments in the group of non-myopic eyes, while we did not find any correlation in the high-myopic eye group; it has been shown that highly myopic eyes have different vascularization characteristics in comparison to eyes without high myopia^18,19^.

We related preoperative structural OCT findings with the final BCVA and in contrast to the results on idiopathic MHs reported in the literature^20–22^. In our casuistry, we found a direct correlation between a larger maximum diameter of MH and final postoperative BCVA. During surgery, we chose the hAM plug diameter based on the pre-operative maximum diameter of the MH; we can hypothesize that a greater quantity of hAM transplanted under the retina leads to better support in the growth of the tissue above the plug, with normalization of DVD parameters, and probably better functional results.

Using Microperimetry, we noticed an increased sensitivity at the hAM plug borders. We consider it a successful outcome, given that these patients had compromised macular conditions, due to the failure of the macular hole. Currently, in the literature, only 1 article includes the microperimetric evaluation after FMH surgery, and in this article, only 1 patient is examined after autologous neurosensory retinal free patch transplantation^23^. The reason probably lies in the fact that the majority of studies have focused on anatomical outcomes.

The Adaptive Optics technology has been used to analyse the status of photoreceptors after idiopathic macular hole surgery by Ooto^24^, Yokota^25^, and Hansen^26^ with AO-SLO, and by Markan using AO-FIO^27^. To the best of our knowledge, this is the first study that includes an Adaptive Optics evaluation after surgery for failed macular holes. Markan et al. have evaluated cone spatial distribution comparing eyes after PPV and peeling for idiopathic macular holes and fellow healthy eyes. Our study showed a statistically significant reduction in cell density and an increase in cone spacing at 2° of eccentricity in all 4 quadrants, compared to healthy fellow eyes. We must consider that in our study, we evaluated eyes that have undergone at least 2 surgeries, where retinal photoreceptors have been subjected to more than 1 surgical stress; we have therefore considered the quantitative evaluation of a low value. Thus, we focused mainly on the qualitative analysis of the images. Using AO-FIO, we found hyper-reflective dots at the edges of the plug, which the integrated software interpreted as a mosaic of photoreceptors. Some larger hypo-reflective dots were found in the center on the edge of the plug, perhaps corresponding to RPE patches or macrophagic cells. This leads to the hypothesis of an initial remodeling process, perhaps triggered by the presence of the hAM plug (Fig. 2).

The usefulness of the hAM plug in closing failed macular holes has already been shown^7^. In our study, we have confirmed the previously published results, including a wider series of patients and introducing new imaging methods to better investigate functional and structural outcomes, and the modifications triggered by the presence of the hAM plug. Not only a complete anatomical success but also a remarkable BCVA improvement and an increase in macular sensitivity have been reached; tissue growth with the formation of layers and signs of remodeling at the retinal capillary plexus level and the cellular level have been found, using the most advanced imaging methods available today. We think that a longer follow-up would be useful for evaluating a further increase in sensitivity in both the peripheral area and the center area of the plug using Microperimetry, and also to evaluate further morphological changes at the vascular plexus level using OCT-A, as well as at the photoreceptor level using Adaptive Optics.

At the end of the follow-ups, we found no differences between the data of patients tamponed with gas and those tamponed with air. We consider it a successful outcome, with the idea of continuing to use only air for future surgery in order to avoid any complications related to the use of gas.

Further and larger studies are necessary to better evaluate the hAM plug potential for the treatment of failed macular holes and the possibility of using it in the treatment of other retinal diseases.

## Methods

This is a retrospective, consecutive, non-randomized interventional study of 36 patients affected by failed macular hole (FMH), treated with 23- or 25-gauge PPV and hAM plug transplantation by 2 expert vitreoretinal surgeons (S.R. and T.C.) at the Eye Clinic of Careggi University Hospital (Florence, Italy) between August 2017 and September 2019. Written informed consent for participation was obtained from all the patients; the study adheres to the Declaration of Helsinki and was approved by the ethical committee of Careggi University Hospital of Florence.

Thirty-six eyes of 36 patients affected by FMH were enrolled in the study. Seventeen eyes were highly myopic (refractive error of at least − 6.00 D or/and axial length ≥ 26.0 mm).

Inclusion criteria were FMH already subjected to PPV with ILM peeling and gas tamponade and follow-up of at least 6 months. Exclusion criteria were the presence of other associated retinal pathologies.

Table 1 summarizes the patient demographics and baseline clinical data.

Patients were examined at baseline and at 2 weeks and 1, 3, and 6 months after surgery. Before surgery, each patient underwent a complete ophthalmologic examination including assessment of best-corrected visual acuity (BCVA), slit-lamp biomicroscopy, intraocular pressure (IOP) evaluation with Goldmann applanation tonometry, axial length assessment with IOL Master (Carl Zeiss Meditec, Dublin, CA, US), OCT angiography and spectral-domain structural OCT (AngioVue Optovue, Fremont, CA, US) analysis with the measurement of the MH basal diameter and MH inner maximum diameter (minimum diameter) and ultra-wide retinography (Daytona, Optos Inc., Marlborough, US).

During each follow-up, patients underwent a complete ophthalmologic examination including BCVA assessment, slit-lamp biomicroscopy, IOP measurement, and spectral-domain OCT (AngioVue Optovue, Fremont, CA, US); at 6 months, ultra-wide retinography (Daytona, Optos Inc., Marlborough, MA, US) and OCT angiography (AngioVue Optovue, Fremont, CA, US) were performed, the latter also in the fellow eye. Adaptive Optics (rtx 1, Imagine Eyes, Orsay, France) and Microperimetry MP3 (Nidek Technologies, Padua, Italy) were performed at 6 months in the group of 19 patients without high myopia.

For the OCTA, the scanning area was captured in 3 × 3 mm sections and was centered on the fovea. Superficial capillary plexus (SCP) and deep capillary plexus (DCP) were automatically segmented by the software. Optovue software of the FAZ function and the density function was used to measure the FAZ area and parafoveal vascular density (VD) of the SCP and DCP. These parameters were measured in FMH eyes and in fellow eyes as well.

The Microperimetric data were analyzed morphologically. Goldmann III in MP3 stimulus size and 4-2 fast-macula threshold strategy was used. The stimulus was projected onto a white background, for a duration of 200 ms. Sensitivities were expressed on the decibel (dB) scale from 0 to 34. For assessment of fixation, the fundus movements were tracked during examination while the patient fixated on a given target, in our case, a 2° single red cross.

Adaptive Optics images of the photoreceptor mosaic, including the area of the surgically closed macular hole, were acquired using a commercially available flood-illuminated AO retinal camera (rtx1, Imagine Eyes). The patient was asked to fixate on a yellow cross controlled by the operator, to allow for imaging of different retinal locations. Images were acquired at 2° of eccentricity along the 4 meridians (nasal, temporal, superior, and inferior). Reduced-quality images, including images in patients with high myopia, were discarded. Each image was evaluated both morphologically and quantitatively, and cones were automatically detected by the software provided by the manufacturer (AOdetect Mosaic b13; Imagine Eyes). Cones within the 2° central area (i.e., up to 1° from the center of the fovea) cannot be resolved because of the limit of resolution of the device. Their spatial distribution was analysed in terms of intercone spacing and local cell density, which were evaluated along the edges of the amniotic plug, where greater sensitivity at Microperimetry was detected. The mean value was calculated from the values in all 4 directions. These parameters were measured in FMH eyes and in fellow eyes as well.

### Surgical technique

Patients underwent a 23- or 25-gauge 3-port PPV (Alcon Surgical, Fort Worth, Texas, US) with a 25-gauge chandelier endo-illuminator to facilitate the bimanual maneuvers. The hAM plug was delivered from the Eye Bank of Lucca, Italy and was cryopreserved. A mixture of membrane blue and trypan blue dyes (Membrane-Blue Dual; DORC International, Zuidlan, Netherlands) was used to check the ILM remnants at the posterior pole. No extra peeling maneuvers were necessary for all the patients. The hAM plug was prepared using a 1 mm or 1.5 mm punch (Disposable Biopsy Punch, Kai Medical, Solingen, Germany), depending on the size of the maximum FMH diameter, measured using OCT. When the FMH diameter was less than 1 mm, the plug was cut inside the vitreous chamber using vitreoretinal scissors. The plug diameter choice is essential for the FMH closure and, consequently, to improve the recovery of visual acuity. An hAM plug that is larger than the basal diameter of the macular hole will tend to create folds because of the tissue redundancy. In the early postoperative period, the retinal inward movement will accentuate the folds and lead to an amassing of amniotic membrane material between the edges of the hole. This will lengthen or stop the recovery process. Conversely, an hAM disc that is smaller than the FMH diameter will not be in contact with the FMH edges. This will not induce FMH closure. The pocket to insert the hAM plug between the MH edges and the RPE was performed using an illuminated PIK (Alcon PIK Endoilluminator, Alcon, Fort Worth, Texas, US). The hAM plug was inserted in the vitreous chamber through a valved trocar. The plug was protected inside the tip of the ILM forceps. Once inside the vitreous chamber, the hAM plug was outstretched using 2 vitreoretinal forceps (Grieshaber Revolution End-Grasping ILM Forceps, Alcon, Fort Worth, Texas, US). Then the chorion layer was identified by detecting the sticky side of the plug using the vitreoretinal forceps. The chorion layer tends to remain adherent to the forceps. Often, with high magnification of the microscope, the 2 sides of the plug can be recognized due to the villi of the chorion layer. The hAM plug was inserted through the macular hole under the retina with the stromal layer facing the retinal pigmented epithelium (RPE) to facilitate its adhesion. The adhesiveness of the chorion alone permits the placement of the hAM plug inside the FMH, in contact with the RPE, without the use of perfluorocarbon liquids or viscoelastic substances. Fluid-air exchange (FAX) and trocar removal were performed. In 23 out of 36 patients (63.8%), 20% sulfur-hexafluoride (SF6) (Fluoron GmbH, Germany) was chosen as the endotamponade; 12 out of 23 were highly myopic eyes. In the remaining 13 patients (36.1%), air was injected. The use of air was chosen for patients on hypotonizing therapy, to avoid post-operative pressure complications. If the chorion layer was correctly facing the RPE, no hAM displacement was reported during fluid-air exchange. The patients were asked to maintain a face-down position for the first 5 days after surgery (see supplemental digital content 1).

### Statistical analysis

Statistical analysis was performed using STATA software version 15.1 (StataCorp., College Station, TX, US). Descriptive statistics were used to summarize mean values and standard deviations of all the numerical data. An independent sample T-test was used to compare the ocular parameters between FMH eyes after surgery and fellow eyes. The Pearson coefficient was used to investigate the correlations between variables. A *p* value < 0.05 was considered statistically significant. The statistical analysis was conducted by considering the group of highly myopic patients separately, as high-myopic eyes have shown changes in vascular density as compared to non-high-myopic eyes^18,19^.

## Supplementary information

Supplementary legend.

Supplementary Video 1.
