# Supplementary material for: Human amniotic membrane plug to promote failed macular hole closure
Source: Sci Rep. 2020 Oct 26;10:18264. doi: 10.1038/s41598-020-75292-2 (PMC7588413; doi:10.1038/s41598-020-75292-2)
Supplement: Supplementary file 1 — Supplementary legend. [file 41598_2020_75292_MOESM1_ESM.docx]

Title: Human Amniotic Membrane plug to promote Failed Macular Hole closure

Authors: Tomaso Caporossi, Bianca Pacini, Daniela Bacherini, Francesco Barca, Francesco Faraldi, Stanislao Rizzo

**Video legend: HAM plug transplant in a failed macular hole, surgical technique.** In this video the hAM plug is introduced through a 25 G trocar, using vitreal forceps. The hAM plug was precut with a cutaneous punch. Once in the vitreal chamber, the plug is gently manipulated and inserted into the macular hole, in the subretinal space, the chorion layer facing the RPE. The chorion layer is determined in the vitreal chamber by identifying the sticky side of the plug using vitreal forceps. Intra-operative OCT is performed to check the correct positioning of the plug. Fluid-air exchange is then performed.
